# Iron concentrations in neurons and glial cells with estimates on ferritin concentrations

**DOI:** 10.1186/s12868-019-0507-7

**Published:** 2019-05-29

**Authors:** Anja Reinert, Markus Morawski, Johannes Seeger, Thomas Arendt, Tilo Reinert

**Affiliations:** 1 Faculty of Veterinary Medicine, Leipzig University, An den Tierkliniken 43, 04103 Leipzig, Germany; 2 Paul Flechsig Institute, Liebigstr. 58, 04103 Leipzig, Germany; 3 Max Planck Institute, Stephanstr. 1A, 04103 Leipzig, Germany; 4 Felix Bloch Institute, Linnéstr. 5, 04103 Leipzig, Germany

**Keywords:** Iron, Ferritin, Neurons, Astrocytes, Microglia, Oligodendrocytes, PIXE, Elemental analysis

## Abstract

**Background:**

Brain iron is an essential as well as a toxic redox active element. Physiological levels are not uniform among the different cell types. Besides the availability of quantitative methods, the knowledge about the brain iron lags behind. Thereby, disclosing the mechanisms of brain iron homeostasis helps to understand pathological iron-accumulations in diseased and aged brains. With our study we want to contribute closing the gap by providing quantitative data on the concentration and distribution of iron in neurons and glial cells in situ. Using a nuclear microprobe and scanning proton induced X-ray emission spectrometry we performed quantitative elemental imaging on rat brain sections to analyze the iron concentrations of neurons and glial cells.

**Results:**

Neurons were analyzed in the neocortex, subiculum, substantia nigra and deep cerebellar nuclei revealing an iron level between $(0.53\pm 2)$ and $(0.68\pm 2)\,\upmu \hbox {M}$. The iron concentration of neocortical oligodendrocytes is fivefold higher, of microglia threefold higher and of astrocytes twofold higher compared to neurons. We also analyzed the distribution of subcellular iron concentrations in the cytoplasm, nucleus and nucleolus of neurons. The cytoplasm contains on average 73% of the total iron, the nucleolus—although a hot spot for iron—due to its small volume only 6% of total iron. Additionally, the iron level in subcellular fractions were measured revealing that the microsome fraction, which usually contains holo-ferritin, has the highest iron content. We also present an estimate of the cellular ferritin concentration calculating $133\pm 25$ ferritin molecules per $\upmu \hbox {m}$ in rat neurons.

**Conclusion:**

Glial cells are the most iron-rich cells in the brain. Imbalances in iron homeostasis that lead to neurodegeneration may not only be originate from neurons but also from glial cells. It is feasible to estimate the ferritin concentration based on measured iron concentrations and a reasonable assumptions on iron load in the brain.

## Background

A balanced iron regulation ensures that iron is kept in appropriate states and concentrations to fulfill its essential functions in the brain while its harmful effects remain controlled. Iron is essential as cofactor of numerous enzymes, especially for ATP production, myelination and synthesis of DNA, RNA, proteins and neurotransmitters [1–3]. No other organ than the brain constantly needs readily available iron in a regional, cellular and age sensitive manner [2]. A failure to meet this demand for iron can result in persistent neurological and cognitive dysfunction. On the other hand, increased iron levels and iron accumulations in specific brain regions and cells are hallmarks for numerous neurodegenerative diseases, but also for the aging brain [1–7]. The prominent neurodegenerative diseases with an iron-associated pathology are Parkinson’s disease and Alzheimer’s disease, but there is also Huntington’s disease, Friedreich’s ataxia, multiple sclerosis, progressive supranuclear palsy, and the group of diseases referred to NBIA (neurodegeneration with brain iron accumulation). An overview of more than 15 diseases is given by Dusek et al. [8].

Oxidative stress is suggested to be a key pathological feature of neurodegeneration and aging processes. Excessive free iron increases the risk to generate highly reactive radicals such as hydroxyl radical via the Fenton reaction. This stimulates oxidative stress and causes damage to DNA, proteins, lipids and can finally lead to cell death.

Nevertheless, the “too much” of iron, usually visualized with magnetic resonance imaging (MRI) scans in individuals with neurological disorders, is not a simple statement per se. The iron metabolism probably differs between neurons, astrocytes, oligodendrocytes and microglia, as each of these cell types has distinct metabolic and architectural features. Thus, the detected iron in the effected brain regions may not be increased in general, but is rather caused by iron accumulations and redistributions associated with certain cell types.

However, the spatial resolution of non-invasive techniques like MRI does not permit the identification of specific cell types [9]. The numerous studies that investigate brain iron and the potential neurotoxicity of iron are mostly comparative studies and/or are based on data obtained with semiquantitative techniques. Semiquantitative techniques either require the addition of chelators (Perls’ and Turnbull’s stain, ferrozine) or fluorescent probes to colorimetrically or photometrically analyze iron [10, 11], or they require sample homogenation (mass spectrometry, electron spin resonance spectroscopy, atom absorption spectroscopy) [12] which precludes analyses with spatial or cellular resolution. The number of techniques that are able to quantitatively determine the “natural” concentration of iron (and other elements) of single cells is limited. Reviews are given by McRae et al. [13] and Bourassa and Miller [14].

Besides the availability of quantitative methods, our knowledge about the brain iron metabolism lags behind our knowledge about systemic iron metabolism [9]. Thereby, a better understanding of the brain iron homeostasis would help to find the causes of the potentially pathological iron-accumulations in diseased and aged brains. With our study we want to contribute closing the gap by providing quantitative data on the concentrations and distributions of iron in neurons and glial cells in situ. A technique that meets the requirements for this task is quantitative elemental imaging with a nuclear microprobe that uses mega-electronvolt protons for scanning particle-induced X-ray emission microscopy ($\mu$PIXE).

## Results

### Qualitative elemental analysis of subcellular fractions of brain homogenate

**Fig. 1:**
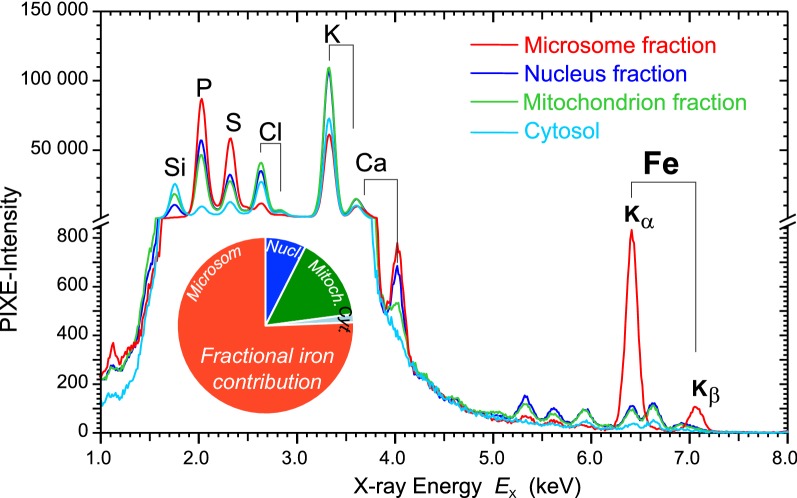
PIXE spectra of subcellular fractions. Particle induced X-ray emission spectra (charged-normalized) acquired from equal amounts of subcellular brain fractions of a neocortex from one rat. X-ray energy ($E_{\mathrm {X}}$) is specific for each element and thus used for elemental identification ($E_{\mathrm {X}}$ of Fe: K$_\alpha$-line: 6.4 keV, $\hbox {K}_\beta$-line: 7.1 keV). The PIXE intensity peak area is a measure for the element concentration. The iron concentration of the microsome fraction is much higher than of the other fractions (peak height of $\approx$ 800 vs. $\approx$ 100). The pie chart inset gives the relative contribution of each fraction to the total iron content of the neocortex homogenate

Fractions enriched in subcellular organelles (nucleus fraction, mitochondrion fraction, and microsome fraction) of neocortex were obtained by differential centrifugation. The contents of the elements P, S, Cl, K, Ca, Fe in the fractions were compared among the different fractions using their PIXE intensities (peak areas) in the charged-normalized PIXE spectra (Fig. 1).

The specific energies $E_{\mathrm {X}}$ of the X-ray fluorescence peaks of Fe are 6.4 keV ($\hbox {K}_\alpha$) and 7.1 keV ($\hbox {K}_\beta$).

The nucleus and mitochondrion fractions have a relative iron content of 8% and 15%, respectively. The relative iron content of the microsome fraction is 75%. The microsome fraction also shows the highest content for P, S and Ca, but the lowest concentration for Cl. The content of Cl and K are highest in the nucleus and mitochondrion fractions. The remaining supernatant of the differential centrifugation, the cytosolic fraction, contains the lowest relative ion concentration of 2%.

### PIXE quantitative elemental imaging of neurons

**Fig. 2:**
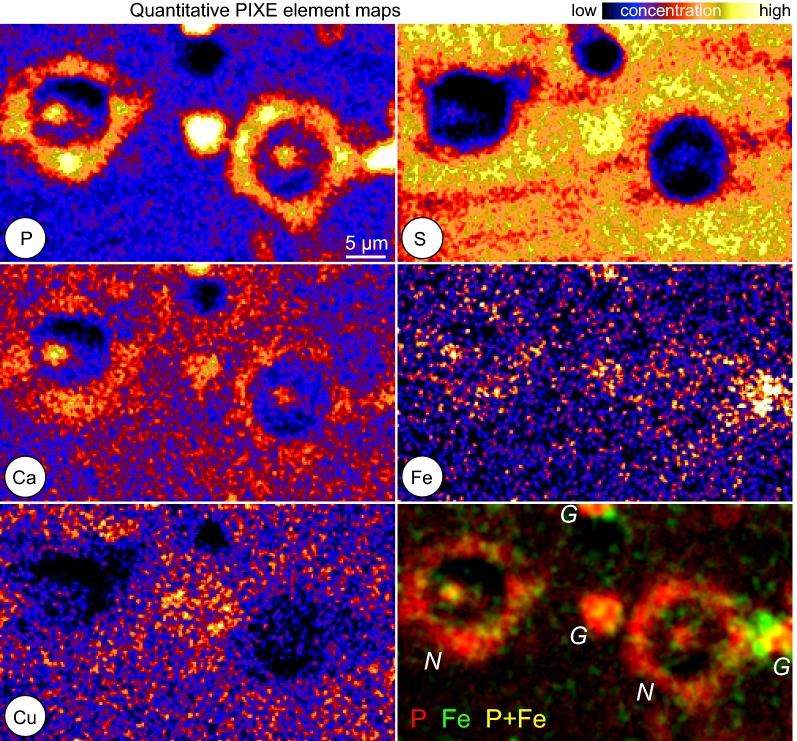
Quantitative element maps. Element images of P, S, Ca, Cu, and Fe. The element images were used to extract the average concentrations from regions of interest. The somata of the neurons are visible due to their high phosphorus content (P image). The extracellular matrix is rich in sulfur (S image). Within the region of the neurons (*N*), the iron map shows elevated iron level in the somata and the hot spot in the nucleolus. The three bright phosphorus spots (top, center, and right in the P-map) are glial cells (*G*) with high iron concentrations

Neurons were identified in the element images by their P-rich cell somata. The high phosphorus concentration (Fig. 2 P-image) is due to the RNA-rich ribosomes.

The sulphur image (Fig. 2 S-image) mainly shows the distribution of the macromolecules of the extracellular matrix that are rich in sulphate groups.

**Fig. 3:**
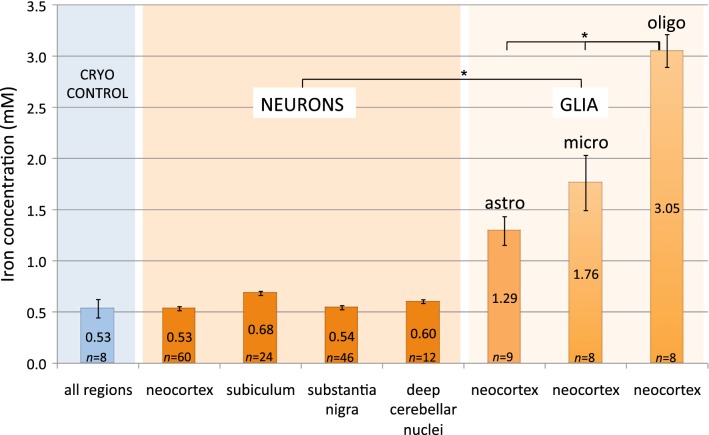
Diagram of intracellular iron concentrations. Intracellular iron concentration (mM) of neurons and glial cells (astro = astrocytes, micro = microglia, oligo = oligodendrocytes) in different brain regions. Control measurements of the investigated regions in native, untreated cryosections proofs that the iron concentration is not effected by the preparation/staining procedure. *n* = number of cells. Values are given as mean ± SE. Statistics: Q–Q-plot, *t*-test. Significance: *$p<0.001$

We quantified the somatic iron concentrations of 142 neurons in total, whereby only neurons distinctly showing a nucleus were selected. We differentiated between neurons in the neocortex ($\textit{n}=60$), subiculum ($\textit{n}=24$), substantia nigra ($\textit{n}=46$) and deep cerebellar nuclei ($\textit{n}=12$). The average iron concentrations of the neurons are $(0.53\pm 02)\,\hbox {mM}$, $(0.68\pm 02)\,\hbox {mM}$, $(0.54\pm 02)\,\hbox {mM}$, and $(0.60\pm 02)\,\hbox {mM}$, respectively (Fig. 3).

In native, untreated cryosections the regional iron concentration is $(0.58\pm 09\,\hbox {mM}$. This shows, that the amount of iron that is possibly washed out during the preparation/staining procedure of the paraffin sections is insignificant for the results and can be neglected.

### Neurons: iron concentrations in cytoplasm, nucleus and nucleolus

**Fig. 4:**
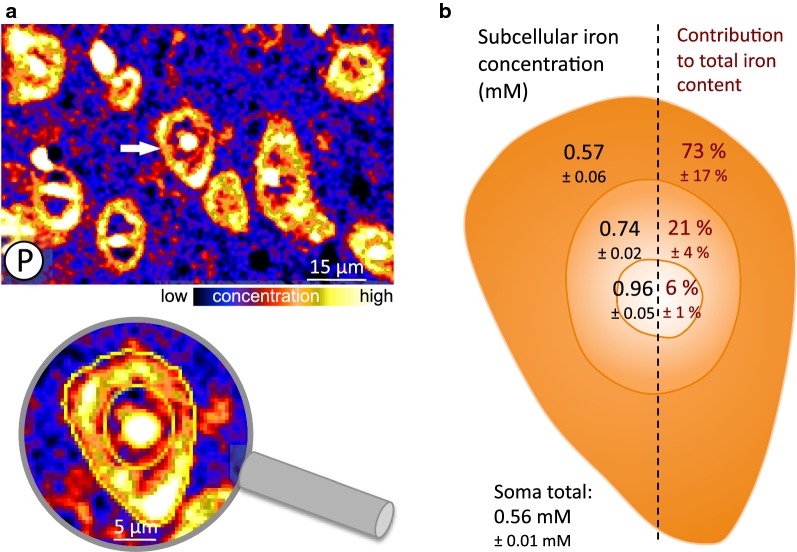
Intracellular distribution of iron in neurons. **a** The phosphorus image (P) was used to set regions of interest (ROI, yellow lines in the zoom image) for the subcellular quantification of iron in neurons. Nucleoplasm appears less P-rich than cytoplasm and nucleolus. The image shows subcortical neurons. **b** Schematic neuron representing (left) the iron concentration (mM) in the cytoplasm, nucleus and nucleolus and (right) the contribution (%) of the compartments to the total iron content of the cell soma calculated for their percentage volume in the cell. Calculation includes neurons from the neocortex, substantia nigra, subiculum, and deep cerebellar nuclei. Number of cells: cytoplasm = 14, nucleus = 19, nucleolus = 50. Values are given as mean ± SE

We further analyzed the iron concentrations of the subcellular compartments cytoplasm, nucleus, and nucleolus. The ROIs for the analysis were set in the P-image (Fig. 4a).

Because only neurons clearly showing both, a nucleus and a nucleolus, neurons of all investigated regions were pooled to increase the related data set (Fig. 4).

**Fig. 5:**
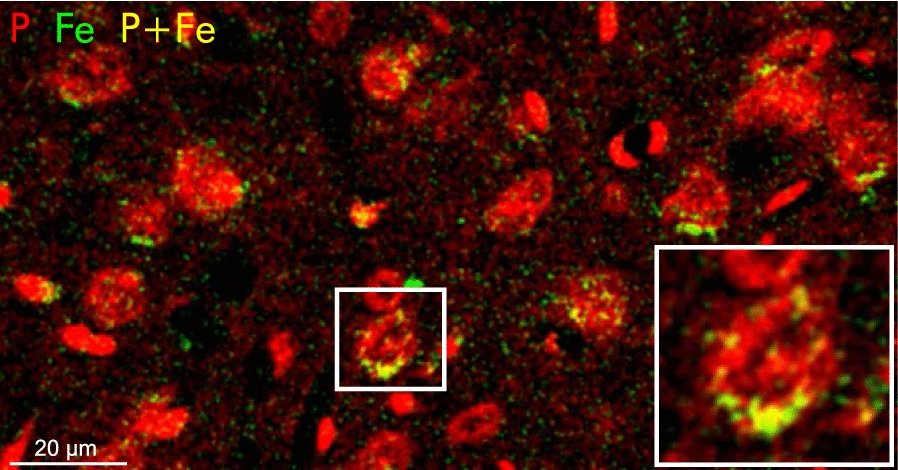
Local iron deposits in neurons. Two element image (red: phosphorus, green: iron) of neurons and glial cells. The enlarged region shows local iron deposits in the cytoplasm of a neuron

The subcellular iron concentration of an “average neuron” is shown in Fig. 4b with iron concentrations of $(0.57\pm 06)\,\hbox {mM}$ in the cytoplasm, $(0.74\pm 02)\,\hbox {mM}$ in the nucleus, and $(0.96\pm 05)\,\hbox {mM}$ in the nucleolus. We further calculated the relative volume of the analyzed compartments to determine the ratio of the iron content of the cytoplasm, nucleus and nucleolus to the total iron content (Fig.  4b values to the right). Most of the cellular iron, $(73\pm 17)\%$, is contained in the cytoplasm. The lowest contribution comes with $(6\pm 1)\%$ from the nucleolus, due to its small volume. Besides the different iron contents in cytoplasm, nucleus and nucleolus, several neurons contained hot spots of iron within the cytoplasm as seen in Fig. 5.

Such iron-rich cytoplasmic sites has been also observed in Perls’ stain (data not shown).

### Glial cells: iron concentrations in astrocytes, oligodendrocytes, and microglia.

**Fig. 6:**
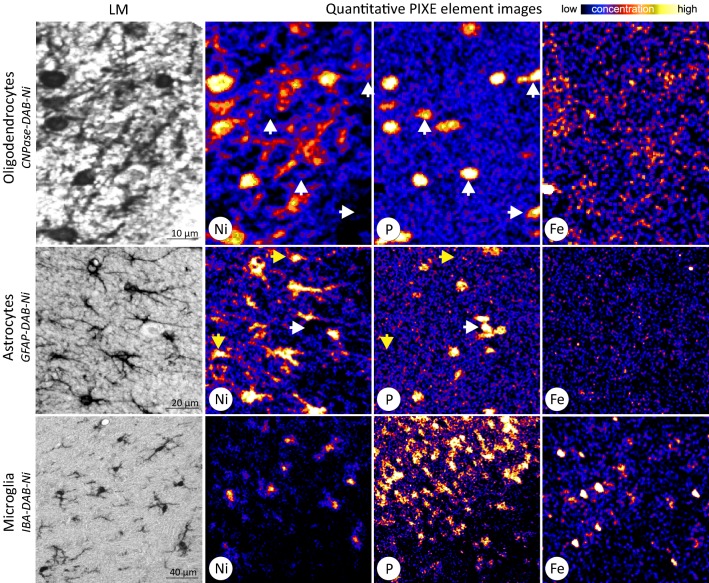
Element maps of glial cells. Light microscopy (LM) and element images (Ni, P, Fe) of the rat cortex specifically stained for oligodendrocytes, astrocytes, and microglia. The staining is enhanced with DAB-Ni (black in LM and high concentration in Ni) to identify and visualize the glial cells by elemental imaging. Glial iron concentrations were extracted from regions of high Ni concentration, which corresponds to the stained glial cells. White arrowheads in the oligodendrocyte and astrocyte images indicate somata of other neuronal/glial cells than the targeted ones (P-rich indicates glial cell, but Ni-negative excludes the target cells). Yellow arrowheads in the astrocyte images point to astrocytes where the somata, shown by the high Ni concentration, but not the nuclei (P-rich) were contained in the $5\,\upmu \hbox {m}$ thin brain section

Astrocytes, oligodendrocytes, and microglia were identified with specific antibodies (see Methods section) and visualized due to their 3,3$^\prime$-diaminobenzidine (DAB)-Ni-labeling seen in the Ni-image (Fig. 6). The Ni-labeling is not only necessary to select specifically the type of glial cells, but also to highlight their soma. In contrast to neurons the high phosphorus region in glial cells is restricted to the nucleus. The cytoplasm cannot be delineated in the P-image. The P-image can therefore not be used to define glial somata. Specific staining that also highlights the soma as seen in the Ni-image is required (Fig.  6 yellow arrows in the astrocyte images).

The average intracellular iron concentration is $(1.29\pm 14)\hbox { mM}$ for astrocytes, $(1.76\pm 27)\hbox { mM}$ for microglia and $(3.05\pm 16)\hbox { mM}$ for oligodendrocytes (Fig.  3). Comparing the iron concentration of the different glial cell types, astrocytes and microglia do not differ significantly, whereby oligodendrocytes’ average iron concentration is significantly higher ($p<0.001$).

### Iron concentration: neurons versus glial cells

The three types of glial cell, analyzed in the neocortex, contain generally more iron than neurons of any analyzed brain region ($p<0.001$, Fig. 3). The average iron concentration of oligodendrocytes is fivefold higher than that of neurons ($p<0.001$). Astrocytes have a twofold and microglia a threefold higher iron concentration than neurons ($p<0.001$).

## Discussion

### Iron concentrations of neurons and glial cells

The neuronal and glial iron concentration was measured in situ and thus represents a reasonable measure for the physiological state. There are only a few quantitative studies on the cellular iron concentrations in the brain. Ortega et al. [15] used synchrotron radiation X-ray fluorescence (SRXRF) to quantify the iron content in a dopamine producing rat cell line (PC12) in vitro. The cell bodies of the cells contained about $(5.0\pm 17)\,\hbox {ng/cm}^{2}$ iron (mean ± SE; *n* = 4–6) when cultured in iron free medium. Under excess iron condition, i.e. adding the subtoxic concentration of $0.3\,\hbox {mM FeSO}_4$ to the medium, the PC12 cells contained about $20\hbox { ng/cm}^{2}$ in their cell bodies (mean ± SE; *n* = 4–6). In our in situ study of neurons the iron concentration of $(0.56\pm 02)\hbox { mM}$ converts to $(15.6\pm 6)\hbox { ng/cm}^{2}$ (mean $\pm \,\hbox {SE}$; $\textit{n}=142$), which falls between the iron depleted and excess limits observed by Ortega and coworkers.

Hare et al. [16] used laser ablation inductively-coupled plasma mass spectrometry (LA-ICP-MS) with the high resolution of $5\,\upmu \hbox {m}$ to analyze the iron content in mice dopaminergic neurons in the substantia nigra and in the ventral tegmental area in situ. They report on iron levels of around $12\,\upmu \hbox {g g}^{-1}$. Since they do not present the concentration in units of millimolar, a conversion is estimated based on the given information on the sample preparation: room temperature dried cryosections of $30\,\upmu \hbox {m}$ thickness. For dried cryosections of $30\,\upmu \hbox {m}$ thickness we have measured (for rat brain) a matrix mass of $0.9\,\hbox {mg/cm}^{2}$. With these assumptions Hare and coworkers’ result of about $12\,\upmu \hbox {g g}^{-1}$ of mice neurons converts to $0.06\,\hbox {mM}$. This is one order of magnitude lower than our results for rat neurons.

Our in situ measurements revealed that oligodendrocytes have the highest iron concentration among the investigated brain cell types. This quantitative data (Fig. 3) are in line with the histochemical findings that oligodendrocytes are noticeably the most iron-rich cell type in the brain [17, 18]. It is assumed that the synthesis and maintenance of myelin, the major function of oligodendrocytes [19], requires high iron levels to cover enzymatic and metabolic needs. Furthermore, oligodendrocytes are likely to mediate iron regulation and are shown to be well equipped with iron storage and transport proteins [20]. This correlates with the high iron content measured in these cells. Oligodendrocytes play a neuroprotective and supportive role or, in case of dysfunction, might initiate or progress the degeneration process (for review see [21]). For example, in mice deficient for iron-responsive element, a model of neurodegeneration due to abnormal iron regulation, oligodendrocytes show a multi-fold increase in ferritin amount while surrounding degenerating neurons have a multi-fold decrease [22].

For astrocytes, Hoepken et al. [23] used atomic absorption spectroscopy (AAS) to measure the iron content in lysate of cultured rat astrocytes. They used Dulbecco’s Modified Eagle’s Medium (DMEM), which contains $0.25\,\upmu \hbox {M}$ iron in the form of ferric nitrate. Normalized to the protein content they received $(9.3\pm 5)\hbox { nmol}_{\mathrm{iron}}/\hbox {mg}_{\mathrm{protein}}$ (mean $\pm \hbox { SE}$; $\hbox {SD}=1.2\hbox { nmol}_{\mathrm{iron}}/\hbox {mg}_{\mathrm{protein}}$, $\textit{n}=6$ cultures). This compares well in the order of magnitude with our in situ results of $(5.9\pm 8)\hbox { nmol}_{\mathrm{iron}}/\hbox {mg}_{\mathrm{protein}}$. For the conversion we need to rely on an estimate of the protein content and cellular mass density. We used for the protein content 20% by weight [24] and a $1.1\,\hbox {g/cm}^3$ for the density. An alternative way for the conversion of our result uses the average cytosolic volume of cells in astrocyte-rich cultures, which has been estimated by the 3-*O*-methylglucose method to be $(4.1\pm 1)\,\upmu \hbox {L/mg}_{\mathrm{protein}}$ [25]. Therewith, our result for the astrocytes’ iron content converts to $(5.3\pm 6)\hbox { nmol}_{\mathrm{iron}}/\hbox {mg}_{\mathrm{protein}}$. Another study on the iron content of astrocytes in vitro, which were derived from newborn mice, was published by Bishop et al. [26]. Using a ferrozine-based colorimetric assay they calculated, based on protein content and estimated cytosolic volume, an intracellular iron concentration of $(1.2\pm 6)\hbox { mM}$ (mean $\pm \,$ SD; *n* ≥ 3 cultures). Within the margin of errors, the iron concentration of mice primary astrocytes published by Bishop and coworkers is the same as we measured for rat astrocytes in situ $((1.29\pm 14)\hbox { mM})$.

However, due to the lack of knowledge on the cytosolic volume of neurons and microglia, [26] were not able to calculate the iron concentration for these cell types. Though, they found microglia and astrocytes to accumulate more iron than neurons, whereby microglia was most efficient. Comparing this with our results where microglia have the tendency ($\textit{p}<0.07$) to contain more iron than astrocytes, and that neurons have the lowest iron content, similarities are obvious. In our data, neurons in neocortex, substantia nigra, subiculum, and deep cerebellar nuclei show the same iron concentration, but glial cells do not only contain more iron than neurons, but also show a different iron concentration depending on the type of glial cell. Therefore, we speculate that the iron concentration depends more on the neural cell type and on the metabolic characteristics and functions of the cell than on the brain region the cell is located in.

Indeed, even that brain cells express a similar set of iron proteins, the amount of each protein expressed varies greatly and depends on the cell type and its iron status [9]. There are also specializations in the iron metabolism, like astrocytes express the ferroxidase ceruloplasmin to regulate their iron efflux, whereby oligodendrocytes express hephaestin [27]. Oligodendrocytes synthesize transferrin, but are lacking a transferrin receptor what makes ferritin their main iron source [28].

Tarohda et al. [29] studied age related changes in metal concentrations in several brain regions of wistar rats. They report increasing iron concentrations during postnatal development from P1 to P42 reaching adult levels before P72. These findings suggest that iron related specializations and regulatory circuits have essentially developed within the first two months of age.

Neural cell types are able to individually regulate the distribution and storage of iron according to their particular needs and functions [30]. A cellular iron depletion or overload is post-transcriptionally regulated by cytosolic iron regulatory proteins that bind to iron-responsive elements of the mRNA which alters its translation or degradation, thus controlling the amount of specific iron proteins for the cellular demand. But it is not the cellular regulatory mechanism itself that ensures the cellular iron homeostasis. Also cell–cell interactions play a supportive or even essential role [5].

For example, oligodendrocytes secrete transferrin to other cells, microglia provide iron to oligodendrocytes to obviously ensure their demand of iron [31], and also astrocytes are known to regulate the transport of iron to other cells [32]. Thus, a balanced cellular iron household depends on a regulated intra-as well as intercellular uptake, storage, distribution and release of iron. It is comprehensible how specialized, precise and organized the brain iron metabolism must work, but also how delicate it is. Also with age iron redistributes between various molecular forms (e.g. ferritin, neuromelanin, transferrin, hemosiderin) and the distribution between neurons and glial cells changes, but this redistribution is only partly understood [5].

### Subcompartimental iron content in neurons

The PIXE-analyzed subcellular fractions obtained by differential centrifugation of brain homogenate shows a very high iron concentration in the microsome fraction. Ribosomes, endoplasmic reticulum and vesicles are enriched in this fraction, and about 50% of the cellular RNA [33]. RNA can directly bind iron and thus may have a contribution to the iron level of this fraction [34]. However, most important, also iron-loaded ferritin is enriched in the microsome fraction [33, 35]. Since up to 90% of the cellular iron in the brain is bound to ferritin [36], the data support its well-known function of being the main iron storage protein in the cell.

Mitochondria are sites of synthesis of Fe-S clusters and heme [37, 38]. A dysfunction of the Fe-S biogenesis results in a variety of diseases also affecting the brain with commonly developed iron overload in the mitochondria [38–41]. However, in healthy rat brain we did not find a crucial impact of the mitochondria fraction to the total brain iron content, its iron contribution is outshined by the impact of the holo-ferritin enriched in the microsome fraction (cytoplasmic ferritin). In neurodegenerative diseases like Alzheimer’s or Parkinson’s, mitochondrial ferritin is increased [42, 43]. But since high mitochondrial ferritin levels result in iron deficient phenotypes in the cytoplasm, its level is maintained low in normal/healthy cells [44]. Our results of an unremarkable iron content in the mitochondria fraction of healthy brain are in line with these data indicating a low level of mitochondrial ferritin with no primary function in iron storage.

Our quantitative imaging of neurons in brain slices revealed (Fig. 4), that the nucleolus has a higher iron concentration than the nucleus and the cytoplasm ($\textit{p}<0.01$). The nucleolus was also found to be a hot spot for iron in plant cells and in human and rat neurons [45, 46]. We can corroborate these observations for mammalian brain cells and now provide quantitative data for the iron concentration in rat neurons.

The nucleolus is the site for ribosomal RNA (rRNA) synthesis and the assembly of the ribosomal subunits (overview in [47]). Besides this, the nucleolus has a very high concentration in RNA, but also proteins and DNA [48, 49]. Thus, its structure is of high optical density in conventional optical microscopy. This high density is also reflected in quantitative elemental imaging where the nucleolus shows high concentrations of elements like P, S and Ca. Since the nucleolus is densely packed and iron can be a co-factor of nucleolar proteins, or associated with rRNA-binding sites, iron is expected to be concentrated too. Roschzttardtz and coworkers even speculated that iron may be involved in the metabolism of rRNA [45].

Additionally, ferritin binds DNA [50] and concentrates iron also in the nucleolus. Another contributing source might be iron directly bound to RNA. The binding capacity of rRNA is higher than for mRNA or tRNA (shown for Fe^2+^ by Honda et al. [34]) which leads to higher iron concentrations in the nucleolus. For Alzheimer’s disease it is shown that rRNA is oxidized by bound redox-active iron [34], i.e, the nucleolus might particularly be vulnerable to oxidative activity causing neuropathology.

There are also conspicuous local iron deposits in the cytoplasm of some neurons. The origin was not further investigated, but cytoplasmic accumulations of iron are known to occur within lysosomes, since they play a key role in iron metabolism and the recycling of iron from e.g. mitochondria and ferritin [51].

### Estimation of ferritin concentration

A ferritin molecule is able to store up to 5000 iron atoms [52]. However, under physiological conditions in the human cerebral cortex and cerebellum ferritin was found to bind approximately 1500 and 1850 iron atoms, respectively [53].

Based on our data of neuronal iron concentration, one can estimate the concentration of ferritin molecules in the cytoplasm with a reasonable assumption of the average number of iron atoms per ferritin molecule with the following equation.

1 $$\begin{aligned} n_{\mathrm {Ft}}\left[ \textstyle \frac{1}{\upmu \hbox {m}^3}\right] =\frac{f\,c_{\mathrm{Fe}}[\hbox {mM}]\,N_{\mathrm{A}}}{N_{\mathrm{Fe}}}\cdot 10^{-18} \end{aligned}$$

*f* is the fraction of iron bound to ferritin (0.9), $c_{\mathrm {Fe}}$ the iron concentration in milli-molar, $N_{\mathrm {A}}$ the Avogadro constant, and $N_{\mathrm{Fe}}$ the average number of iron atoms per ferritin molecule. The factor $10^{-18}$ results from unit conversions to calculate the ferritin concentration $n_{\mathrm{Ft}}$ in units of molecules per cubic micrometer. Assuming a load of 2400 iron atoms per ferritin molecule (midrange of data from [22]) and taking the average neuronal iron concentration of $0.59\hbox { mM}$ (Fig. 3), we calculate 133 ferritin molecules per $\upmu \hbox {m}^{3}$ in rat neurons with an uncertainty of $\pm \,25$ (based on estimated uncertainties of 0.1, $0.06\hbox { mM}$, and 250 for *f*, $c_{\mathrm{Fe}}$, and $N_{\mathrm{Fe}}$, respectively). This density is in agreement with the results from Zhang et al. [22] who quantitatively analyzed the Fe-load and distribution of ferritin in and around axons in mouse brain slices. They report for wild-type mice the number of iron atoms per ferritin molecule to be in the range from 1200 to 3600 and average densities of 130 and 133 ferritin molecules per $\upmu \hbox {m}^{3}$ (two tomograms analyzed). The concentration was relatively uniform in regions inside and outside axons.

Going beyond comparing our estimate of ferritin concentration with the matching result from Zhang et al. [22], we want to propose Eq. 1 as a reasonable estimation for ferritin concentrations based on measured total iron concentrations and the iron load as a parameter. Table 1 gives, for the here presented results, the estimates of ferritin concentrations in neurons and glial cells for three different ferritin iron loadings.

**Table 1:** Estimated concentrations of ferritin molecules, derived from measured iron concentrations with the iron load parameter $N_{\mathrm{Fe}}$ in Fe atoms per ferritin molecule (Fe/Ft) according Eq. 1

	Fe conc. (mM)	Estimate of ferritin concentration		
		@1200 Fe/Ft ($\upmu \hbox {m}^{-3}$)	@1800 Fe/Ft ($\upmu \hbox {m}^{-3}$)	@2400 Fe/Ft ($\upmu \hbox {m}^{-3}$)
Neurons	0.57	270	180	130
Astrocytes	1.29	580	390	290
Microglia	1.76	790	530	400
Oligodendrocytes	3.05	1380	920	690

### Relevance in neuropathology and therapy

Failures in iron homeostasis mechanisms may be a pathological condition that causes oxidative stress and subsequently neurodegeneration. It is still not clear whether the observed excessive Fe accumulation in the brain is always the initial event or a consequence of the disease process [54, 55]. However, a better understanding of iron accumulation in brain disorders has therapeutic implications. Iron chelators are being explored in pre-clinical models and clinical trials for several diseases associated with brain iron imbalance, including Parkinson’s disease, Alzheimer’s disease, Friedreich’s ataxia and amyotrophic lateral sclerosis [3, 56–59]. Especially promising results in clinical studies were achieved with iron-chelating drugs [60–62]. However, currently, the clinical effect of an iron chelation therapy still remains unclear [9, 58, 62, 63]. Not only the differences among the brain regions and the cell types involved in each disorder makes it unlikely to succeed as a general strategy, but also the cellular differences in iron concentrations and distributions presented in this study suggest differences in functionality and pathological potential. A cell-specific misregulation or mislocation would either result in local anemia and functional deficiency, or in iron-induced oxidative stress [30]. This calls for customized iron chelators that are designed to specifically target only relevant cell types.

## Conclusions

Based on the assumption that the glial cells’ functions and physiology are similar among the brain regions, we conclude that glial cells are the most iron-rich cells in the brain, from which oligodendrocytes have the highest concentration. Any study which measures iron content in the brain with lower than cellular resolution should not only focus on neurons, but also consider glial cell iron contributions. Similarly, imbalances in iron homeostasis that lead to neurodegeneration may not only be localized in neurons but also, or even in the first place, in glial cells.

Since the majority of brain iron is bound to ferritin, it is feasible to estimate the ferritin concentration based on reasonable assumption of the average ferritin loading and measured iron concentrations.

## Methods

For the study two months old Wistar rats (*Rattus norvegicus f. domestica*) were used, three male rats for quantitative mapping and analysis of the intracellular iron, and one female rat for the qualitative analysis of iron in subcellular fractions. The animals were obtained from and housed at the animal care facility of the Paul Flechsig Institute for Brain Research of Leipzig University. They were kept on a 12/12h dark/light cycle with free access to food and water. Experiments were carried out in accordance to the guidelines of the European Council Directive (1986; 86/609/EEC) and with approval by the local authorities. All animals were anesthetized in a 5-L anaesthesia chamber by opening a 100% $\hbox {CO}_2$ influx to a flow rate of 1 L/min. After confirmation of unconsciousness by loss of the tail clamp response the animals were quickly sacrificed by decapitation or perfusion.

### Iron content in subcellular fractions of brain homogenate

#### Differential centrifugation

**Fig. 7:**
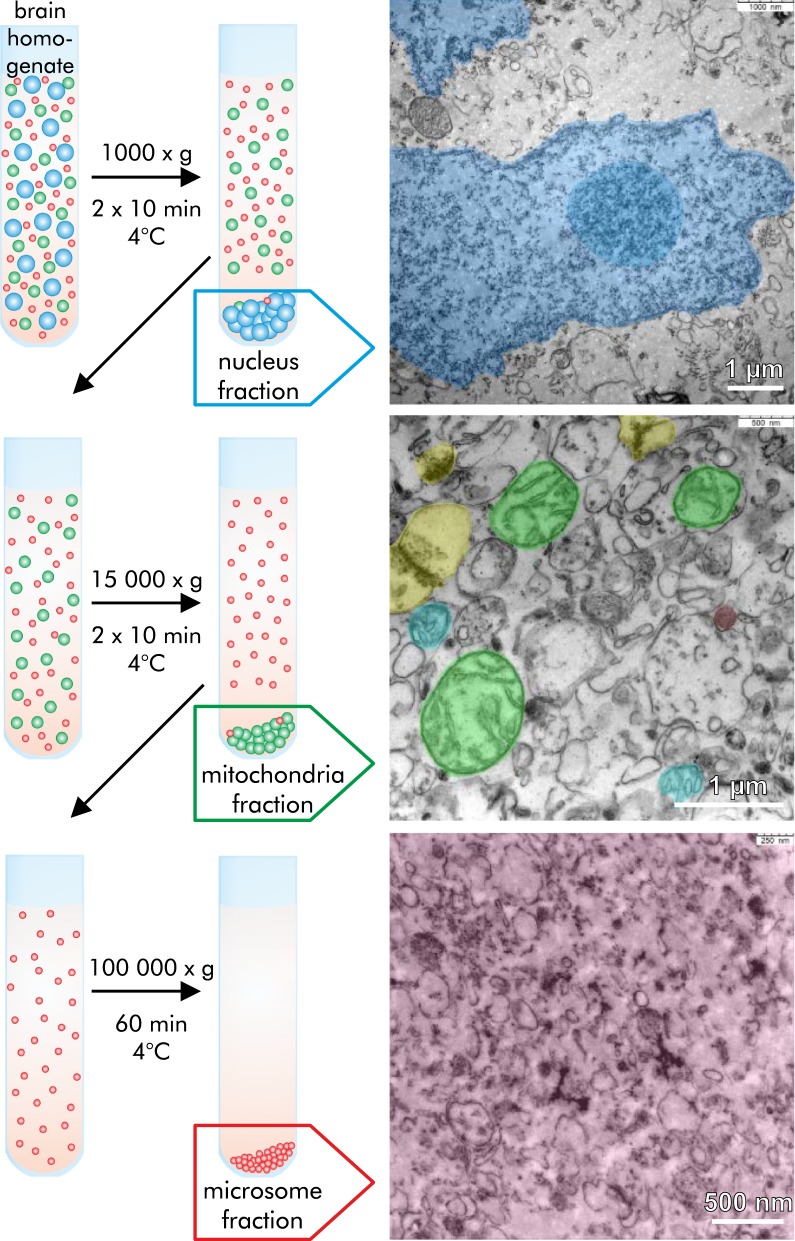
Differential centrifugation protocol. Differential centrifugation protocol for fresh neocortex homogenate in 0.25 M saccharose solution to obtain the following subcellular fractions: nucleus fraction (blue), mitochondria fraction (green) and microsome fraction (red). The enrichment of the target particles is verified by transmission electron microscopy. The nucleus fraction contains cell nuclei (highlighted in blue, nucleolus in darker blue) as well as myelin fragments as seen at the very bottom of the picture. The mitochondria fraction is enriched in numerous mitochondria (highlighted in green), but also synaptosomes (highlighted in yellow), Golgi complexes (highlighted in light blue) and lysosomes (highlighted in red). The microsomal fraction contains microsomes (highlighted in red, summarizing vesicles, plasma membranes, ribosomes and endoplasmic reticulum)

Differential centrifugation was used to separate and enrich subcellular particles from brain homogenate. Therefore, we homogenized one complete snap frozen rat neocortex in 0.25 M saccharose solution (final dilution of 10%) with a Dounce homogenizer on ice. The homogenate was centrifuged (Sorvall Combi Plus, DuPont, USA) according to the protocol given in Fig. 7. For the nucleus and the mitochondria fraction the centrifugation step was performed twice and the first and second pellets were pooled of each fraction. The fractions were verified by transmission electron microscopy.

The following subcellular fractions were obtained: (1) nucleus fraction containing cell nuclei and myelin fragments, (2) mitochondria fraction enriched in mitochondria, synaptosomes, Golgi bodies, peroxisomes and lysosomes, and (3) microsome fraction consisting of plasma membranes, ribosomes, endoplasmic reticulum and vesicles. (4) The supernatant of the differential centrifugation, was referred to as cytosolic fraction.

#### Transmission electron microscopy

$10\,\upmu \hbox {L}$ of each fraction were fixed in 4% formaldehyde and 2% glutaraldehyde in 0.1 M phosphate-buffered saline (PBS, pH 7.4) for 30 min at room temperature, washed twice in Aqua bidest by centrifugation, embedded in 3% agarose gel, contrasted/fixed in 1% osmium tetroxide (Merck) for 1 h at $4\,^{\circ }\hbox {C}$, rinsed thoroughly in PBS, dehydrated in a series of acetone and contrasted with 1% uranyl acetate (Merck) in 70% acetone for 45 min at room temperature. For sectioning the samples were embedded in Durcupan araldite casting resin M (Fluka) according to a standard embedding procedure. Semithin sections (500 nm) were cut on an ultramicrotome (Reichert) and stained with toluidine blue for initial observation. Ultrathin sections (50 nm) were cut and examined by transmission electron microscopy (TEM) (Zeiss LEO 912 Omega and Zeiss Libra 120). All fractions were proven to be enriched in their specific subcellular particles (Fig. 7).

#### PIXE spectrometry

In order to analyze the same volume of each subcellular fraction with particle induced X-ray emission, $5\,\upmu \hbox {L}$ were dropped on a $2\hbox { mm} \times 2\hbox { mm}$ wide and 200 nm thin $\hbox {Si}_{3}\hbox {N}_{4}$ membran (Silson Ltd, UK) and air-dried. A 2.25 MeV proton beam was scanned in a square of $1.6\hbox { mm} \times 1.6\hbox { mm}$ over the center of the dried droplet while the X-ray spectrum was collected and normalized by the charge, i.e. by the number of exciting protons. Since the peak areas of the characteristic X-ray lines are a measure of the elemental contents in the equal-volume droplets, the charged-normalized peak areas were used to calculate the proportion of total iron of the individual subcellular fractions.

### Quantitative elemental imaging of neurons and glial cells in brain slices

#### Preparation of brain sections

Rats (${n}=3$) were sacrificed with $\hbox {CO}_2$ and transcardially perfused with saline (0.9% NaCl)/0.1% heparin to eliminate hem-iron. Further, a fixative solution of 4% formaldehyde and 0.1% glutaraldehyde in 0.1 M PBS (pH 7.4) was transcardially perfused for 30 min.

Brains were removed from the scull, cut into three coronal sections using a scalpel, and post-fixated in the same fixative solution overnight at room temperature. After dehydration in increasing ethanol concentrations and followed repletion in methylbenzoate, the samples were embedded in paraffin. Frontal sections of $5\,\upmu \hbox {m}$ thickness were cut containing the neocortex (Bregma $4.1\hbox { mm}$), subiculum, substantia nigra (Bregma $4.1\hbox { mm}$) and deep cerebellar nuclei (Bregma $10.8\hbox { mm}$). The sections were transferred to Superfrost^®^ glass slides, deparaffinized with xylene, rehydrated in decreasing concentrations of ethanol and transferred into PBS (pH 7.4).

#### Immunohistochemistry

Brain slices for glial cell analysis were immunohistochemically stained at $4\,^{\circ }\hbox {C}$ over night with polyclonal rabbit IgG against glial fibrillary acidic protein (GFAP, AB_10013482, Dako, 1:1000) for astrocytes; ionized calcium binding adaptor molecule 1 (IBA-1, AB_839506, Wako, 1:500) for microglia; or oligodendrocyte-specific protein (OSP, AB_2276205, abcam, 1:200) for oligodendrocytes. Brain slices were washed in PBS (pH 7.4) and were then incubated with the secondary antibody (biotin-conjugated donkey anti-rabbit IgG, AB_2340585, JacksonImmunoRes, 1:1000). Further, all brain slices were washed in PBS (pH 7.4), rinsed in Tris-HCl (pH 8) and were then incubated for 1 h at room temperature with peroxidase-conjugated streptavidin (ExtrAvidin^®^, SCR_013728, Sigma, 1:1000) to reveal the antibody binding sites. The staining was enhanced by 3,3$^\prime$-diaminobenzidine (DAB, Sigma) and Ni (nickel ammonium sulphate, purity grade 99.999%, Sigma) in Tris-HCl (pH 8). Brain slices were finally rinsed in Tris-HCl and PBS again. Ni is used as enhancer, because it is visible with light microscopy due to its black precipitate, and in elemental imaging due to its characteristic X-ray emission [64]. The Ni-staining was proven to not introduce any significant impurities, especially to not alter the distribution and concentration of iron [64–66].

#### Embedding for $\mu$ PIXE analysis

For quantitative elemental imaging by scanning particle induced X-ray emission ($\mu$PIXE), the brain sections, still on Superfrost^®^ object slides, were covered with a small droplet of mounting medium (DePeX, Serva) that was spread out by shortly covering the sections with another object slide. Holding the object slide sandwich vertically, whereby the cover slide was allowed to move freely downward by gravity, a thin layer of embedding medium was produced after the cover slide eventually slipped off. After 24 h of drying at room temperature a rectangular area of $20\hbox { mm}\times 15\hbox { mm}$ containing the brain section was cut out, peeled off, and attached to aluminum frames using double sided adhesive carbon tape. Thus, free-standing $15\,\upmu \hbox {m}$ thick DePeX foils were prepared that had the brain sections embedded, but not covered on the surface later facing the X-ray detector.

Light microscopic images (Olympus BXiS, Germany) of each framed brain slice were done for orientation and re-recognition of the cells within the PIXE element maps.

Additionally, fresh rat brain was snap frozen in liquid nitrogen chilled isopentane to prepare $15\,\upmu \hbox {m}$ thick cryosections (Bregma $4.1\hbox { mm}$) using a cryostat microtome (HM 500 O, Microm). The sections were sandwiched between two formvar foils (Serva) as a support for elemental analysis. The sections were dried at $-\,36\,^{\circ }\hbox {C}$ in the cryostat to prevent potential elemental redistribution caused by thawing.

#### $\mu$ PIXE analysis

Quantitative elemental imaging and analysis were done with a $1\,\upmu \hbox {m}$ proton beam of 2.25 MeV energy using the high energy ion nanoprobe LIPSION at Leipzig University, Faculty of Physics and Geosciences, [66, 67]. The proton beam was scanned over the brain sections while the induced X-rays emitted from the sample were recorded. This technique is called PIXE, the prefix “$\mu$” in $\mu$PIXE refers to the capability of microscopic element imaging using a scanned focused beam. Quantitative analysis is based on (1) spectral deconvolution by least squares fitting of element peaks and background, (2) calculated yield to each element from fundamental parameters for X-ray production and matrix effects, and (3) the theoretical description of the detectors responses, geometric parameters, and absolute efficiencies [68]. The correct description of the detector system is verified by analysis of certified reference standards [69].

From the recorded X-rays, tagged with the position, overlap-free and quantitative element images were created using dynamic analysis [70], which is part of the GeoPIXE software. GeoPIXE also provides a wide range of graphical tools that were used to encircle the regions of interest in the images and determine the average elemental concentrations therein. Since the MeV-protons cause relatively low background radiation in the element characteristic X-ray spectrum, the minimum detection limits, especially for elements of atomic number between $Z = 21\dots 30$, thus also for iron, are at $\upmu \hbox {g/g}$-level which corresponds to concentrations around $10\,\upmu \hbox {M}$.

#### Statistics

The significance of differences between elemental concentrations of neurons and glia cells was tested using the *t*-test with unequal sample size of cells from three rats. The test of common distribution was performed with a Q–Q-plot. $\mu$PIXE data between the three rats did not differ significantly (*t*-test, $\textit{p}<0.05$).

One rat was used for the differential centrifugation. Therefore, no statistical test was performed. The difference between the microsome and the other fractions is with a factor of eight large enough to justify the use of a single rat.

## Data Availability

The datasets analyzed during the current study are not publicly available yet, because results of another aspect are included that we intend to submit later on. However, they are available from the corresponding author on reasonable request.

## Abbreviations

AAS: atomic absorption spectroscopy
DAB: 3,3′-diaminobenzidine
Ft: ferritin
LA-ICP-MS: laser ablation inductively-coupled plasma mass spectrometry
LM: light microscopy
MRI: magnetic resonance imaging
NBIA: neurodegeneration with brain iron accumulation
PBS: phosphate-buffered saline
PIXE: particle induce X-ray emission
$\mu$PIXE: scanning particle induce X-ray emission
SRXRF: synchrotron radiation X-ray fluorescence
TEM: transmission electron microscopy
